# Silver Nitrate in the Treatment of Pulpless Teeth

**Published:** 1899-05

**Authors:** L. G. Noel


### ARTICLE X.

### SILVER NITRATE IN THE TREATMENT OF PULPLESS TEETH.

DR. L. G. NOEL.

For some months past the writer has been experimenting with this drug in the treatment of pulpless teeth, and the results have been so satisfactory that he feels it his duty to lay the matter before the profession. It may be that others are using this means of sterilizing pulp chambers and canals, but so far he has heard nothing of the practice. He writes this, believing it to be the best method for the molar teeth that he has used, and he has tried almost all the remedies that have been suggested for this purpose. It can not be denied that good results may be obtained with many other agents, but it is doubtful if absolute disinfection and sterilization of the dentin can be so quickly obtained with any other drug as with the silver nitrate. Its solubility makes it more penetrating than anything else that has been exploited in this practice; but this quality and its blackening effects upon the teeth, will almost limit its employment to the molars. True it may be used in the treatment of lower bicuspids, and in many places where discoloration has already occurred as a result of chemical changes in pulp tissue, and where it is not deemed especially objectionable its application may be extended to other teeth. Now a word as to the method of applying it. Having cleansed the pulp canals of septic matter as thoroughly as possible, and removed as much of the softened and aseptic dentin from the wall of the pulp-canals as can be taken away with impunity, and having allayed all peridental inflammation to the point of toleration of slight percussion, we put on the rubber dam, and after washing out all other medicaments, we apply the silver nitrate as follows: Having obtained from the druggist a short, wide-mouthed bottle of blue or green glass, containing the finely pulverized crystals, we take a smooth platinoid probe (a gold probe would probably be befter, but good results may be had with steel or platinoid), and, wrapping a fiber of cotton loosely around the point of the probe, it is first dipped into water, then into powdered nitrate, and carried as far into the canal as it will go. The probe may then be withdrawn, leaving the cotton fiber with its charge of silver nitrate behind.

The probe is now used as a plugger, and the cottou pushed as far into the canal as it will go. This process is repeated until the canals are packed as full as they can be, then a pledget of cotton is moistened and rolled in the powdered nitrate and pushed into the pulp-chamber. The cavity of entrance may be closed with some temporary stopping, and the case dismissed for a week or two.

Upon the return of the patient, the whole may be re-removed and the roots filled. Such is the solubility and penetrating power of this agent that we believe it will filter through the dentin and render aseptic any portions of the roots traversed by canals so tortuous or flattened as to elude cleansing instruments of operator. Our faith goes farther. We believe that the cotton (if raw cotton) might be left in the canals with such a charge of silver nitrate, and the permanent filling introduced over it, with as good result as can be obtained by other methods of root-filling. We are not yet prepared to adopt this as a practice, not to recommend it to others, but we have been trying it in cases where it has been impossible to obtain that degree of dryness necessary to make a good root-filling of gutta-percha or oxychlorid of zinc, and so far the results have been very gratifying. In many of the buccal roots of the superior molars, where nothing but a mere fiber of cotton can be placed, we would say saturate this fiber with silver nitrate and leave it, using oxychlorid of zinc, or some more solid filling-material for permanent filling of the palatine canal. Perhaps the most gratifying results that we have yet obtained have been in the treatment of pulpless deciduous teeth. Our method of procedure has been as follows : After opening the pulp chamber and allaying the soreness by the use of dressings of old wood creosote, we fill the pulp-chamber about half full of cotton, moistened and rolled in pulverized silver nitrate. A temporary stopping is placed over this, and it is left for ten days. Upon the return of the patient the dressing is removed, the carious dentin is excavated from the crown of the tooth, and, after syringing out carefully with pasteurine, and bathing it with creosote, the whole cavity is filled with copper amalgam, without any effort at cleansing and filling the pulp-canals. We have not had to tap a single case to relieve alveolar abscess where the above treatment has been used; however, it is a comparatively new practice, and we admit that a few short months is insufficient to establish it. The roots of these deciduous molars being usually greatly wasted by the process of resorption, and the canals always difficult to broach, no one has had much success in cleansing and filling them; therefore we believe all will hail with delight a method that gives success without these baffling efforts. That eminent writer, Dr. Safford G. Perry, has recommended merely cleansing and disinfecting the pulp-chambers; then, if trouble arises after filling, tapping the roots to give exit to gas.

Who has not felt that the boring of a tape-hole was a clumsy way to relieve a very regretable septic condition ? Our silver nitrate cases (and we have thus treated quite a number) have so far exhibited no symptoms of septic trouble. Not the least recommendation for the above practice is the ease and speed with which it may be executed. For the copper amalgam filling, we will say that its greatest value is for the deciduous teeth, and in these cases tt is especially indicated.—*Dental Headlight.*

